# COVID-19 and ethical preparedness?

**DOI:** 10.1007/s00508-020-01709-7

**Published:** 2020-07-08

**Authors:** Christiane Druml

**Affiliations:** UNESCO Chair on Bioethics at the Medical University of Vienna, Ethics, Collections and History of Medicine, Medical University of Vienna, 1090 Vienna, Austria

**Keywords:** COVID-19, Pandemic, Ethics, Fundamental human rights, Infectious diseases

## Abstract

Mankind has to prepare for a pandemic with respect to medical and practical aspects, but also with respect to ethical issues. There are various ethical guidelines for managing infectious disease outbreaks, but they do not apply to the specific aspects of the COVID-19 pandemic, since they were formulated after the different kinds of outbreaks of avian influenza and Ebola. Today we are confronted with completely new issues endangering our fundamental human rights. As COVID-19 is spreading all over the world, we are in a desperate situation to find treatment solutions; however, despite the urgency, scientific rules have to be applied as bad science is unethical since it might be harmful for patients. Fake news and alternative facts might not be easily recognized and are also threatening scientific values. Pandemics might be leading to a meltdown of the health system if no measures are being taken constraining fundamental human rights. Tracking of persons is violating human rights as well if not accepted on a voluntary basis. A failure to have safeguards for times of crisis leads to a scarcity of medicinal products and goods resulting in a nationalistic approach and ignorance of international solidarity. And last but not least selective measures and triage in intensive care have to be taught to young physicians and nursing staff in medical schools in order to be prepared in times of an infectious disease outbreak and scarcity of resources.

Mankind has learned to live with infectious disease outbreaks. History is full of diseases, such as the plague, smallpox or cholera ravaging continents in times where there were no therapies or economic ways to battle them [1]. The medical, economic and social achievements of the past 150 years have made a change. Still, every outbreak is a challenge as if it was the first one. One reason is the nature of man, putting preparations off when menacing scenarios are not an immediate threat. Bill Gates stated some years ago at the Munich Security Conference that he “views the threat of deadly pandemics right up there with nuclear war and climate change”, and “that we need to prepare for epidemics the way the military prepares for war” (https://www.gatesfoundation.org/Media-Center/Speeches/2017/05/Bill-Gates-Munich-Security-Conference). It is obvious that the world has not been guided by the same conviction [2].

A pandemic like the novel coronavirus disease SARS-CoV‑2 changes the perception of our life and decisions in our health systems rapidly. In Italy, where the increase of critically ill patients has been exponential for weeks, intensive care unit (ICU) beds were scarce, and overworked physicians had to decide over life and death [3]. We saw the same developments in other parts of the world, although the pandemic has not affected all countries equally.

There are recent guidelines for managing ethical issues in infectious disease outbreaks, but they are not a practical tool for the physician at the bedside [4] or are relevant only for specific issues, such as research (https://www.nuffieldbioethics.org/publications/research-in-global-health-emergencies). In outbreaks, decisions must be made quickly, even when there is yet no evidence. The challenge is to prepare practical solutions for times of epidemics.

Pandemic is not pandemic. Avian influenza and the most recent outbreak of Ebola, which took place in Africa represent the main background for current ethical guidance documents. Since then, many topics have dramatically changed and we are confronted with completely new and expanding issues, which increase with time and location of the worldwide spread of the coronavirus. One can predict that key issues and ethical guidelines will look much different after the end of the coronavirus pandemic; however, we do need an ethical decision-making framework which guides us in providing systematic and practical answers to ethical questions.

What are the new ethical issues we face?

## Clinical research to find new treatments

The uniqueness of COVID-19 is that it is new and unknown in its properties, highly contagious, and there are no specific drugs nor vaccines to treat the patients or prevent infection. All the identified potential drugs and antivirals have to be screened to find out if they are an option; however, they are not made specifically for the coronavirus, many are not licensed treatments yet. That means they have to undergo a standard clinical trial research program to expand the indications or to get the market authorization. Even in a desperate situation and the given pressure of public and governments for speed as with COVID-19, it is necessary to compare the identified options with the local standard of care in large randomized controlled clinical trials in as many countries as possible to generate valid results quickly and as scientifically sound as possible. Small trials or compassionate use programs will not be able to provide robust data in a comparable time and are thus not giving proper scientific evaluation, which is ethically unacceptable. Bad science is unethical and dangerous since it might be harmful for patients. Drugs, such as hydroxychloroquine have well-documented risks; administering the drug outside of any adequate clinical trial for COVID-19 would be unjustifiable without any clear clinical benefit [5].

## Fake news, alternative facts and myths

Access to clinical information and rapid data sharing is essential. It is a lifesaving necessity, representing a tremendous advantage of knowledge crucial for saving life. But how can we differentiate between reliable information and alternative facts? Fake news might be disguised, can discriminate and harm humans, destroy trust in the medical system and counteract the necessary acts to mitigate or suppress an outbreak. Fake news and myths, not specific for times of crisis, aggravate the situation [6]. The internet and social media are its breeding ground. How can we recognize them?

## Constraints of fundamental human rights

We do not have effective drugs against the coronavirus, and the first weeks of this outbreak in Wuhan showed that the death toll was rising, a meltdown of the health system needed to be avoided. Governments had to resort to means as old as civilization in times of an epidemic, namely isolation and quarantine of persons to protect the vulnerable and to preserve the health system, in particular ICUs. Isolation and quarantine, the restriction of free movement are all measures breaching fundamental human rights. A breach which is only acceptable if it is based on the rule of law and observes the principle of proportionality, if only the less severe means are to be used. Governmental decisions raise concern regarding the relationship between the liberty of individuals and societal needs. All restrictions have to be limited for the time of crisis and obligatorily they have to be lifted as soon as the outbreak is over [7].

## Social media and tracking of persons

Governments try to flatten the curve of contagion to avoid a meltdown of the health care system by requiring the population to stay at home, to practice social distancing.

Smart phones allow persons to be tracked and to observe if persons keep their quarantine. In some countries from early on tracking of persons via the smartphone provider is being practiced [8]. This poses a breach of the fundamental right of privacy and acceptance depends on the voluntariness or the obligation of the use of the relevant smartphone application. A great part of the population accepts this intrusion understanding that one has to act, but how will we be assured that the fundamental human rights are reestablished once the crisis is over?

## Scarcity of medicinal products and goods—the nationalistic approach “my country first”

Another feature we can see is the nationalistic approach to and withdrawing from international solidarity. In a world, where international trade is a global mantra, a pandemic leads to a sudden critical shortage of goods, such as test kits and protective gear [9]. There are few producers worldwide and they act according to the motto “everybody for himself” while frontiers are closed.

The recent past has already shown dependency on vital drugs. China produces 80% of the heparin used worldwide. African swine fever has diminished the supply chain leading to highly reduced swine population and low quality of heparin [10]. Although in the European Union a great number of companies have concessions for production, profit was perceived to be too low to manufacture.

Where are the safeguards to hinder shortcomings in times of crisis?

## Selective measures in intensive care and triage—managing ethical dilemmas

General ethical principles are well known, but there is considerable ignorance about the specific applicability in crisis. Many of us live in countries offering a public health system where nobody questions the boundless availability of high-tech measures and interventions. Germany and Austria are the countries with the highest per capita number of ICU beds. But the sheer amount of patients flooding the hospitals in countries like Italy, Spain or France requiring intensive care treatment made us aware that we needed to prepare ethical guidelines for scarce resources and triage. Times of a pandemic are not the place for starting capacity building and training in this field. Curricula of medical schools have an obligation of teaching specific knowledge. Furthermore (young) physicians and nurses do have to train in their daily work deliberations for difficult decision making. Decisions for which they are not prepared in the sudden situation of an outbreak, especially when they have not trained their intellectual and emotional capacity in this field in good times to have them at hand in bad times [11].

## Conclusion

The general ethical principles are always the same, namely justice and solidarity, beneficence, non-maleficence and autonomy. Ethical preparedness is as relevant as the provision of surgical masks or swabs, or the formulation of relevant laws to encounter urgencies in the field of public health. Let us keep this in mind, also after the end of this pandemic, whenever it may come. In a slight change of that what Bill Gates said in Munich: “The fact that a global pandemic has (…) occurred in recent history shouldn’t be mistaken for evidence that a deadly pandemic will not occur (again) in the future”.
